# Platelet rich clots are resistant to lysis by thrombolytic therapy in a rat model of embolic stroke

**DOI:** 10.1186/s13231-014-0014-y

**Published:** 2015-01-27

**Authors:** Amelia J Tomkins, Nadine Schleicher, Lucy Murtha, Manfred Kaps, Christopher R Levi, Max Nedelmann, Neil J Spratt

**Affiliations:** School of Biomedical Sciences & Pharmacy, University of Newcastle, and Hunter Medical Research Institute, Newcastle, Australia; Heart and Brain Research Group, Justus-Liebig-University, Giessen and Kerckhoff Clinic, Bad Nauheim, Germany; Department of Neurology, Justus-Liebig-University, Giessen, Germany; Department of Cardiac Surgery, Kerckhoff Clinic, Bad Nauheim, Germany; Hunter New England Local Health District, Newcastle, Australia; School of Medicine and Public Health, University of Newcastle, and Hunter Medical Research Institute, Newcastle, Australia; Sana Regio Klinkum, Pinneberg, Germany; Department of Neurology, University Hospital Center Hamburg-Eppendorf, Hamburg, Germany

**Keywords:** Embolic Stroke, Microbubbles, Platelet Rich Clot, Rat, Thrombolysis, Ultrasound, Sonothrombolysis

## Abstract

**Background:**

Early recanalization of occluded vessels in stroke is closely associated with improved clinical outcome. Microbubble-enhanced sonothrombolysis is a promising therapy to improve recanalization rates and reduce the time to recanalization. Testing any thrombolytic therapy requires a model of thromboembolic stroke, but to date these models have been highly variable with regards to clot stability. Here, we developed a model of thromboembolic stroke in rats with site-specific delivery of platelet-rich clots (PRC) to the main stem of the middle cerebral artery (MCA). This model was used in a subsequent study to test microbubble-enhanced sonothrombolysis.

**Methods:**

In Study 1 we investigated spontaneous recanalization rates of PRC *in vivo* over 4 hours and measured infarct volumes at 24 hours. In Study 2 we investigated tPA-mediated thrombolysis and microbubble-enhanced sonothrombolysis in this model.

**Results:**

Study 1 demonstrated stable occlusion out to 4 hours in 5 of 7 rats. Two rats spontaneously recanalized at 40 and 70 minutes post-embolism. Infarct volumes were not significantly different in recanalized rats, 43.93 ± 15.44% of the ischemic hemisphere, compared to 48.93 ± 3.9% in non-recanalized animals (p = 0.7). In Study 2, recanalization was not observed in any of the groups post-treatment.

**Conclusions:**

Site specific delivery of platelet rich clots to the MCA origin resulted in high rates of MCA occlusion, low rates of spontaneous clot lysis and large infarction. These platelet rich clots were highly resistant to tPA with or without microbubble-enhanced sonothrombolysis. This resistance of platelet rich clots to enhanced thrombolysis may explain recanalization failures clinically and should be an impetus to better clot-type identification and alternative recanalization methods.

**Electronic supplementary material:**

The online version of this article (doi:10.1186/s13231-014-0014-y) contains supplementary material, which is available to authorized users.

## Background

Currently, the only approved thrombolytic treatment for acute ischemic stroke is recombinant tissue plasminogen activator (tPA) delivered intravenously within 4.5 hours of stroke onset [1]. However, less than 10% of all ischemic stroke patients are eligible for therapy [2] and tPA achieves successful recanalization in less than half of those treated [3]. Achieving higher recanalization rates in a timely manner is a key goal to developing better stroke thrombolytic therapy.

Use of ultrasound to enhance recanalization with tPA is a promising approach. The first clinical report of stroke sonothrombolysis indicated increased rates of recanalization in patients receiving continuous transcranial Doppler ultrasound (TCD) monitoring during tPA therapy [4]. Several small clinical trials with perflutren-lipid and galactose-based microbubbles as enhancers of sonothrombolysis suggest that microbubbles may produce further improvements in the rates of recanalization [5-8]. Despite the promise of this therapy, more than half of patients treated do not recanalize [9] and concerns have been raised regarding its safety with increased rates of hemorrhage in some studies [6,10]. Therefore, there is still a great need for pre-clinical studies to better understand the efficacy, mechanisms and safety effects of this potential therapeutic strategy.

To test any thrombolytic therapy, a model of stroke is required that uses a life-like clot to block major cerebral arteries. Current thromboembolic models are highly variable and it is likely that this variability is related to the choice of clot and its inherent stability with regards to spontaneous and thrombolytic-induced lysis. Recent studies have demonstrated that platelet-rich clots (PRC) are more stable *in vitro* than other clot variations and are also more similar to clots retrieved from human stroke patients histologically [11]. These clots have yet to be tested *in vivo* and during sonothrombolysis.

In this article we describe a method of embolic stroke using site-specific delivery of a PRC to the origin of the middle cerebral artery (MCA). Two separate studies were performed. In study 1 our aim was to investigate rates of spontaneous recanalization and infarct volumes using our new PRC model. In study 2 we aimed to investigate the effect on recanalization rates of tPA therapy alone or in conjunction with ultrasound and a new microbubble formulation (BR38) in this model.

## Methods

### Animals

All animal experimentation was carried out in accordance with local legislation. Experiments conducted at the University of Newcastle, Australia (**UoN**) were in accordance with Animal Care and Ethics Committee (ACEC) guidelines (approval # A-2010-128) and in compliance with the requirements of the Australian Code of Practice for the Care and Use of Animals for Scientific Purposes. Experiments conducted at Justus-Liebig University Giessen, Germany (**JLU**), were in accordance with the German animal protection legislation and approved by the regional ethics committee (Az. B2/257). At **UoN**, spontaneously hypertensive rats (SHR) aged 14–18 weeks and weighing 320-375 g, were sourced from Animal Resources Centre, Perth, Australia. At **JLU**, 15–21 weeks old SHR weighing 290-375 g were sourced from Harlan laboratories GmbH, Netherlands. Surgeries were performed at both centres by the same investigator (AT). Anaesthesia was induced with 5% isoflurane and maintained at 1.5-3% in oxygen and nitrogen (2:1). Temperature was monitored and maintained at 37°C throughout all experiments. SpO_2_ and heart rate were also monitored at **UoN** (Study 1).

### Experimental design

Study 1 was performed to determine spontaneous recanalization rates in the first 4 hours post-embolism, and 24 hour infarction volume and mortality (n = 14; performed at **UoN**). In Study 2 we investigated the effect of tPA or tPA + ultrasound + BR38 microbubbles compared to saline control in this model (n = 44; performed at **JLU**). The specifics of each protocol are outlined in Table 1.

**Table 1:** **Experimental protocols**

	**Study 1**	**Study 2**
**Location**	Newcastle, Australia	Bad Nauheim, Germany
**Aim**	Develop model, Determine spontaneous recanalization rate	Determine recanalization rates with microbubble + sonothrombolysis enhancement of tPA
**Primary Outcome [method]**	Recanalization [Laser Doppler]	Recanalization [Laser Doppler]
**Secondary Outcome(s) [method]**	Infarct Volumes [TTC], Mortality, Neurological Deficit scores	Clot lysis [Inspection of the major branches of the cerebral arterial circulation]
**Survival post-embolism**	24 h	2 h
**Treatment groups (n)**	No treatment (n = 7)	Saline (n = 10), tPA (n = 10), tPA + Ultrasound + BR38 microbubbles (n = 10)
**Laser Doppler Monitoring**	Continuous	Discontinuous
	*To 4 h post-embolism*	*Pre-embolism, Post-embolism, Pre-treatment, Post-treatment*

Clot preparation was modified from the methods of Roessler *et al.* [11]. Briefly, cardiac blood from donor animals (Study 1: n = 4, Study 2: n = 10) was collected with sodium citrate anticoagulant (3.2%; blood:citrate = 9:1) and underwent 2-step centrifugation to obtain platelet rich plasma. Whole plasma and buffy coat were collected after an initial spin (15 minutes at 180 *g*). The platelet-rich plasma layer and buffy coat were collected after a second fast spin (20 minutes at 1500 *g*). PRC formation was initiated by recalcification of the platelet rich plasma + buffy coat with CaCl_2_ (20 mM final concentration). The sample was drawn into a 40 cm length of PE-50 tubing (40 cm, i.d. 0.58 mm) and clamped at one end. Using an air filled syringe, pressure was forced into the unclamped end of the catheter to create clots of consistent size. This end was then also clamped to retain the pressure and incubated at 37°C for 2 hours. The resultant clot was ejected into a dish of saline and stored at 4°C overnight. Clot preparation was the same for both studies, with the exception for Study 2 in which the clot was incubated for 5 minutes in Evans blue for visualisation within major branches of the cerebral arterial circulation at sacrifice.

The method of site-specific clot delivery was based on the method of MCA thread occlusion we use routinely [12-14] with catheter placement and clot delivery as described by DiNapoli *et al.* [15]. Briefly, a modified catheter (PE-8, o.d. 0.35 mm, connected to silastic tubing) containing a 30 mm length of PRC was inserted into the internal carotid artery via the external carotid artery. It was advanced until a sensation of mild resistance indicated the tip was at the middle cerebral artery (MCA) origin, then retracted 1–2 mm to restore flow to the MCA allowing site-specific clot injection. The clot was injected with 30–50 μl saline and the catheter left in place for 10 minutes to allow the clot to stabilize. The catheter was then completely removed from the vessel and the surgical site sealed.

### Confirmation of occlusion and recanalization rates

In both studies, confirmation of occlusion of the MCA after clot injection was made by laser Doppler Flowmetry (LDF). Occlusion was defined as a reduction to < 50% from baseline values of regional cerebral blood flow (rCBF). The primary outcome of both studies was recanalization rates. Recanalization was defined as a return to ≥ 100% of baseline rCBF. Two methods of LDF monitoring were used: continuous and discontinuous.

### Study 1

Continuous laser Doppler tissue perfusion monitoring was performed using a single fibre optic probe (MoorVMS-LDF with probe type VP10M200ST, Moor instruments, UK). A longitudinal incision was made along the scalp, above the midline and over bregma. The skull was thinned using a dental drill 1 mm posterior to bregma and as far lateral as possible (at the border of the temporalis muscle). The Doppler probe was affixed to the skull within a silicone probe holder. The position of the probe was inspected under the operating microscope before fixation to ensure it was not placed over large vessels that would otherwise compromise the accuracy of the readings. The recordings were taken for a minimum 20 minutes pre-embolism. Baseline rCBF was calculated as the average of a 5 minute period immediately prior to clot injection. All subsequent readings were expressed as a percentage of baseline. Continuous LDF monitoring also allowed confirmation of the initial MCA occlusion by the clot delivery catheter, followed by catheter retraction (restoring flow) and clot injection. LDF monitoring continued for the duration of observation under anaesthesia (4 hours post-embolism). Four hours of post-occlusion monitoring was based on the approved time window for clinical tPA administration to determine if these clots were stable during that period. Clinical benefit of thrombolysis beyond this time is limited [16]. Animals were then woken and returned to their cages until sacrifice at 24 hours for infarct volume analysis.

### Study 2

Continuous monitoring could not be performed during ultrasound treatment for Study 2 due to the apparatus set-up, so a discontinuous approach was used [17]. Discontinuous monitoring was performed at 4 time points: pre-embolism (−30 minutes), post-embolism (10 minutes), pre-treatment (50 minutes) and post-treatment (130 minutes), using an Oxyflo2000 Microvascular Perfusion Monitor with Oxyflo XP Probe 17 mm diameter (MNP 100XP-3/15, Oxford Optronix, UK). Recordings were made as per Soehle *et al.* [17]. Animals were sacrificed after final LDF recording for visual inspection of clot presence.

### Treatment groups

Animals in Study 2 received intravenous injections via the tail vein of either tPA, tPA and microbubbles, or vehicle (saline) started 1 hour after embolism (n = 10 per group). The tPA and microbubble group also received 60 minutes ultrasound. tPA (10 mg/kg; Actilyse™, Boehringer Ingelheim, Ingelheim, Germany) was delivered as a 10% bolus and the remainder delivered over 1 hour. Total injected volume was 2.4 ml. The dose of 10 mg/kg was used based on evidence that the rat fibrinolytic system is 10-fold less sensitive than humans [18]. A total of four 0.1 ml doses of BR38 microbubbles (Bracco Research, Switzerland) at a concentration of 4 × 10^8^ bubbles/ml (10 μl BR38 diluted in 90 μl NaCl), were delivered at 15 minute intervals starting with the initial tPA bolus. BR38 are 35% perfluorobutane and 65% nitrogen in a phospholipid shell. The dose of BR38 was comparable to previously calculated doses of other microbubble formulations [19]. Transcranial colour-coded Duplex ultrasound (TCCD) was applied continuously for the duration of treatment (60 minutes). A 3 MHz diagnostic ultrasound probe was placed 40 mm above the skull (B-mode, color-Doppler functions switched on, maximum output (mechanical index of 1.7)) (Sonos 7500; Philips Ultrasound, USA). The distance between the skull and ultrasonic probe was bridged with ultrasound gel (Sonosid®; Asid Bonz, Germany) in a plastic cylinder open at both ends and the beam was aligned to expose the entire brain incorporating the circle of Willis and the occluded MCA (spectral Doppler sample volume placed in the midbrain (57 mm)).

### Additional outcomes

For both studies, the primary outcome was LDF recanalization. Secondary outcomes were infarct volume, neurological deficit and mortality for Study 1, and visualization of PRC in the MCA post-mortem for Study 2.

For animals surviving 24 hours post-occlusion (Study 1), a series of neurological tests were performed to determine level of functional deficit following stroke. Deficit was assessed by an observer blinded to recanalization outcome with a modified Bederson scoring system [20,21], scoring the degree of flexion of the affected limb, degree of twisting of the animal’s torso, and the ability to brace against a lateral force. Each test was given a score of 0, 1, or 2 where 0 = not affected and 2 = severely affected. These scores were totalled for a final score between 0 and 6. Mobility was also scored, 0 = mobile and 2 = immobile.

Infarction was assessed at 24 hours in Study 1. Animals were sacrificed by isoflurane followed by cardiac perfusion with cold saline (3 minutes, Peri-Star™ Pro perfusion pump, World Precision Instruments, USA). Brains were removed and placed in cold saline (4°C), stirred gently with a magnetic stirrer. Brain slices (2 mm) were covered in 1% 2,3,5-triphenyl-tetrazolium chloride (TTC) solution and incubated at 37°C for 10 minutes. Slices were turned and covered in fresh TTC for a further 10 minutes at 37°C, stored overnight at room temperature in buffered formalin then photographed. Hemispheres and regions of infarct were traced using ImageJ software and infarct volumes were calculated by averaging the area of the top of the section and the area of the base of a section and multiplying by the width (volume of a trapezoid). The total infarct volume was the sum of all slices. Infarct volumes were expressed as a percentage of the ipsilateral hemisphere.

**Figure 1:**
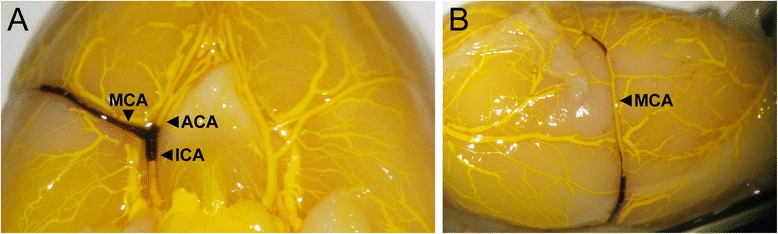
**Visualisation of vascular filling and clot presence (Study 2).** Vessels were perfused post-mortem with Microfil (yellow) to visualise the vasculature and clot presence (black). All animals had clot in the major cerebral vessels after treatment. **(A)** Shows the vasculature from the view of the Circle of Willis, **(B)** shows the lateral surface of the right hemisphere. Vessels labelled are: middle cerebral artery (MCA), anterior cerebral artery (ACA) and internal carotid artery (ICA). Images of all brains can be viewed in the Additional file 1.

Arterial filling and clot visualisation in Study 2 was achieved by intravascular silicone infusion (Figure 1). Animals were sacrificed immediately following post-treatment LDF (130 minutes), and Microfil (Flow Tech, Inc., USA) was used to cast the vasculature, as previously described [19]. Briefly, the circulation was flushed with saline until the venous effluent was clear of blood. The descending aorta, subclavian arteries and both left and right external carotid arteries were ligated and Microfil, prepared to manufacturer’s instructions, was injected via the aortic arch to fill the arterial and venous cerebral circulation via internal carotid and vertebral arteries. During the injection process, excessive dilation of the aorta was avoided so as to maintain physiological pressure conditions. After 45 minutes, the Microfil formed an elastomeric gel at room temperature and the brain was removed from the skull and immersed in formalin. The Circle of Willis and the lateral surfaces of the brain were photographed for visualisation of clot presence.

### Inclusion/exclusion criteria

Pre-specified exclusion criteria were as follows: No appropriate LDF drop after clot injection (Study 1 & 2); spontaneous recanalization pre-treatment (Study 2); Subarachnoid hemorrhage (SAH) due incorrect catheter insertion was identified during continuous LDF monitoring (Study 1) or post-mortem (Study 1 & 2). Excluded animals in study 2 were replaced so that treatment groups contained 10 animals.

### Statistical analysis

Data is presented as mean ± standard deviation (SD). Differences in infarct volumes between recanalized and non-recanalized animals were assessed with unpaired Student’s *t*-test. Statistical significance was considered to be a p-value < 0.05.

## Results

### Study 1 – Investigation of the Model

Three animals were excluded from analysis due to either SAH (n = 1) or occlusion not confirmed by LDF (n = 2). SAH was identified by LDF signal and confirmed post-mortem. One animal died overnight post-embolism. Post-mortem histology revealed large infarction to be the likely cause of death. This animal was included in LDF analysis, but not infarct calculations or neuroscoring.

**Figure 2:**
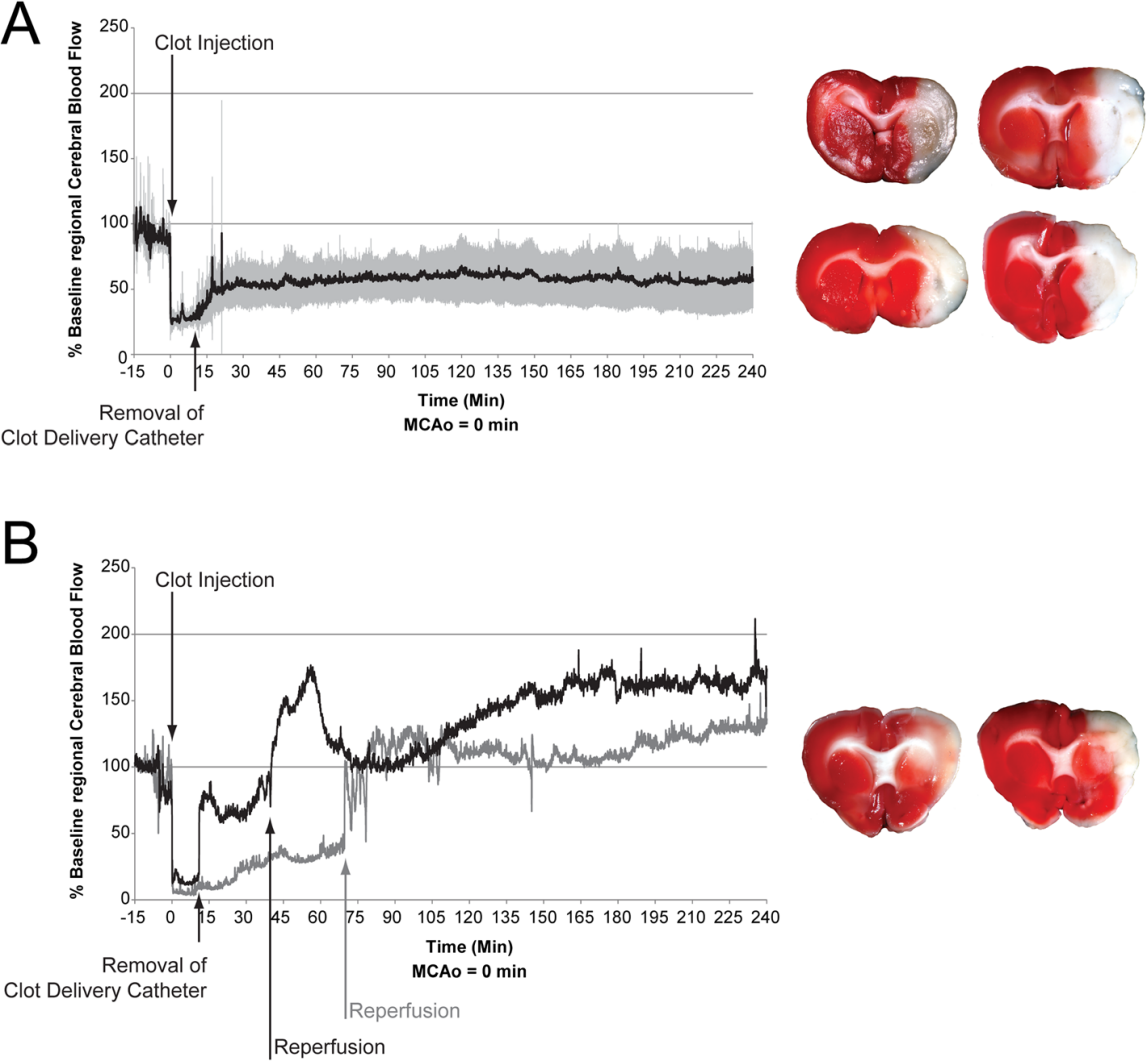
**Laser Doppler flowmetry and infarction following middle cerebral artery occlusion with platelet rich clots (Study 1).** A total of seven rats had successful embolization of the MCA. **(A)** Five rats remained occluded for the duration of LDF observation (mean ± SD). One rat died overnight and was not included in TTC assessment of infarct. **(B)** Two rats recanalized at 40 min (black trace) and 70 min (grey trace) (raw data).

Five of seven animals remained occluded for the duration of LDF monitoring (4 hours post-embolism) (Figure 2A). Two animals spontaneously recanalized at 40 and 70 minutes post-embolism (Figure 2B). Infarct volumes were 48.93 ± 3.9% and 43.93 ± 15.44% of hemisphere in non-recanalized and recanalized rats, respectively. There was no statistical significance between groups (p = 0.7), however the rat that recanalized earlier (40 minutes) had a smaller infarct than the one that recanalized later (70 minutes): 33.01% v. 54.85% of hemisphere, respectively. Neuroscores at 24 hours were 3 ± 2. All animals exhibited a neurological deficit.

### Study 2 - Thrombolytic treatment effect

**Figure 3:**
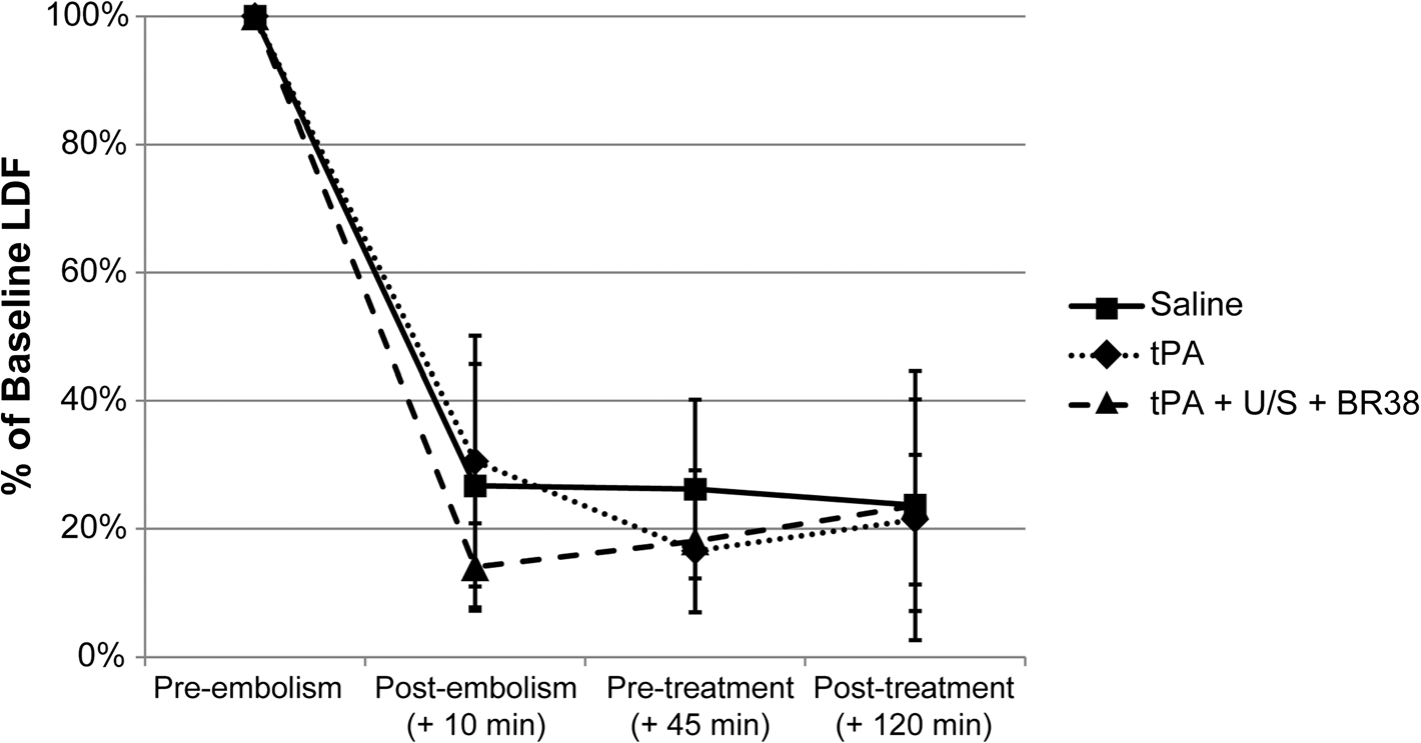
**Laser Doppler flowmetry (LDF) of regional cerebral blood flow in treatment groups (Study 2).** Animals underwent embolization of the middle cerebral artery (MCA) with platelet rich clot. LDF confirmed occlusion in all animals and indicated no recanalization post-treatment. Data represents the mean of n = 10 per group. There were no significant differences between groups. (U/S = ultrasound; BR38 = microbubbles).

A total of 4 animals were excluded due to SAH (n = 2, determined post-mortem), excessive bleeding pre-embolism (n = 1), and catheter dislodgement during treatment (n = 1). These animals were replaced so that all groups consisted of 10 animals. No animals recanalized in any treatment group in Study 2 (Figure 3 and see Additional file 1). Clot was visible within the Circle of Willis of all animals except one in the ultrasound group, however clot was observed in the distal MCA of this animal (Figure 1 and see Additional file 1).

## Discussion

We present here an experimental model of embolic stroke using PRC. This model was developed because clots retrieved from patients often have high platelet concentrations [22-25], unlike most commonly used experimental clots, which are red cell or fibrin-rich [11]. We have shown the experimental PRC to be highly resistant to spontaneous and tPA-mediated thrombolysis with or without enhancement with microbubble-enhanced sonothrombolysis. This high resistance to lysis suggests that greater levels of platelets may be an important contributing factor to failed recanalization in many stroke patients despite tPA treatment.

Our findings confirmed PRC resistance to thrombolysis with tPA, and indicate that clots enriched with platelets are highly resistant to lysis, even with the addition of ultrasound and microbubbles. Within the fibrin network of a PRC clot, large platelet aggregates are observed. These aggregates lead to increased rigidity of the clot and significantly increase time to tPA-induced lysis compared with other clots [11,26]. Histological comparisons of experimental PRC with clots retrieved from human stroke patients have also revealed both to contain a strong cross-linked fibrin mesh that ensures mechanical stability [11]. The addition of platelets to thrombin-induced clots also increases their resistance to thrombolysis *in vivo* [27]. A major inhibitor of plasminogen activation is plasminogen activator inhibitor (PAI-1) released locally by platelets and vascular endothelial cells. PAI-1 has been implicated as increasing PRC resistance to tPA lysis [28,29].

There are several putative mechanisms for the action of sonothrombolysis. These include microstreaming of blood, that enhances access of tPA into the clot, and mechanical disruption of the fibrin mesh [30]. Our results showed that microbubble-enhanced sonothrombolysis was insufficient to lyse thrombi highly enriched in platelets and this may be an important consideration when delivering reperfusion therapy to patients. While sonothrombolysis appears to enhance recanalization rates in stroke patients, there is a subset of patients (33–59%) who do not achieve complete recanalization despite the addition of sonothrombolysis with or without microbubbles [6,8,9,31]. Clot composition in this subgroup is not known, however from studies of mechanically retrieved thrombi from stroke patients we do know that they are very heterogenous and high platelet levels occur frequently in clinical stroke [22-25]. Moreover it has been shown, that clot composition is correlated with outcome after 90 days [32]. Our data suggest a likely explanation for at least some of these patients with failure of recanalization is PRC resistance to thrombolysis. If this is the case, alternative approaches will be needed.

Stroke is a heterogeneous condition with no two clots compositionally the same [24] thereby resulting in differing efficacies of tPA thrombolysis. The majority of preclinical *in vivo* studies do not directly compare clot types. Investigating only one clot type is also a limitation of our study. However, *in vitro* work of PRC has shown greater resistance to thrombolysis than other clot types [11,26], with a recent study demonstrating increased tPA resistance with increasing platelet counts [33]. PRC are also better histological mimics of clinical thrombi retrieved from stroke patients who failed to recanalize after tPA therapy. Our study confirms PRC resistance to tPA thrombolysis in an *in vivo* rat model and also demonstrates resistance to enhanced sonothrombolysis. Studies of sonothrombolysis in rat models are surprisingly lacking. Most have used low frequency ultrasound or were not performed in embolic models. Studies of 10 mg/kg tPA thrombolysis in rat embolic MCA occlusion models are the closest comparisons we can achieve, and only a few report recanalization. Spontaneously formed clots with high erythrocyte content as well as thrombin induced-fibrin rich clots recanalize in >80% of cases [34-36]. These experimental clots may mimic the clots of tPA responders, but does not allow room for testing thrombolytic enhancers. Since no recanalization was observed in our study, it is reasonable to conclude that the composition of our clots has a large effect on thrombolysis. The lack of preclinical studies comparing clots, or studying sonothrombolysis at diagnostic frequencies clearly highlights a gap in our knowledge of the full impact of clot type on sonothrombolysis *in vivo*. It should also be noted that even with microbubble-enhanced sonothrombolysis, a large proportion of patients still do not respond to therapy [37]. Our study could provide one answer to this – that the occluding thromboemboli of these patients contain high proportions of platelets.

An important implication of these findings is that there may be a subset of patients with PRC and even the combination of ultrasound with tPA may be largely futile to cause recanalization. The clinical risk of haemorrhage with increasing tPA doses prevents testing higher doses to determine if thrombolytic recanalization can be achieved at all in this clot type. The dose of tPA we used (10 mg/kg) is already a high dose, with studies of other clot types suggesting that the clinical dose is a better mimic of the clinical response to recanalization [38]. Further increasing our tPA dose would unlikely exhibit significant increases in recanalization and a more clinically relevant interpretation of these results is that PRC do not lyse solely by the fibrinolytic mechanisms initiated by tPA and ultrasound. Early identification of patients with clots of higher platelet content might allow time to pursue alternative approaches to reperfusion, such as mechanical clot retrieval. Another consideration is the use of antiplatelet agents. Both GPIIb/IIIa receptor blockers (abciximab) and cyclo-oxygenase inhibitors (aspirin) have shown promise in reducing clot lysis time and causing decreases in platelet-fibrin aggregates when applied prior to clot formation. Reduced clot lysis time has been observed when abciximab is added to pre-established clots [26], suggesting a possible therapeutic strategy. There is certainly promise in using combination antiplatelet -tPA therapy, however there is clinical concern regarding the risk of additional bleeding complications in stroke patients [39,40]. Several imaging approaches have been used to try and identify resistance of clots to thrombolysis. MRI and CT studies of experimental and clinical stroke clots have begun to correlate signal intensities with composition of red cells and fibrin [23,25,41-43]. So far, studies have been able to identify red cell rich clots from other clot types, due to the presence of iron in these cells that increases signal intensity. Clots high in red cells were shown to be more easily lysed [11]. However the current study indicates that platelet content may be a key factor affecting recanalization rates. As yet, the ability to distinguish fibrin-rich from platelet-rich clots is limited.

A limitation of this study is that we did not assess haemorrhage, a known complication of sonothrombolysis [37]. Clinical evidence suggests that TCCD may cause higher rates of haemorrhage than TCD monitoring [37]. We have previously demonstrated efficacy of microvascular recanalization using this system and parameters, and with ultrasound. Intracerebral haemorrhage was observed in only 1 of 25 animals treated with tPA + ultrasound + microbubbles, and this protocol was deemed safe for sonothrombolysis [19]. All clinical sonothrombolysis studies using TCCD have used ultrasound insonation durations of 60 minutes [31,44,45], which was our choice for this study. Yet the majority of TCD studies have insonated for 2 hours [6,7,9,46]. Increasing insonation duration may result in greater recanalization rates, but this direct comparison has not been investigated clinically and it is unknown what the optimal duration is for maximal efficacy with minimal adverse events. Given the clinical evidence of increased haemorrhage with TCCD, any study of longer duration insonation should consider safety as an important outcome.

Our ability to determine the efficacy of BR38 microbubbles to enhance sonothrombolysis was limited in this study. BR38 microbubbles were developed to improve on the existing SonoVue™ formulation and are more stable and circulate longer in the microcirculation [47]. A previous study showed that BR38 enhanced sonothrombolysis of spontaneously formed thrombi [48], a relatively easy to lyse clot type [49,50]. Our decision not to include a sonothrombolysis group without microbubbles was made because such experiments with diagnostic frequencies and BR38 microbubbles have not been performed in rats. There is sufficient evidence that microbubbles have greater efficacy than ultrasound + tPA alone [37]. Therefore, we chose to determine maximal effect with these bubbles, while limiting animal numbers for ethical reasons. Had an effect between groups been observed, it would have warranted further study to determine the exact effect of these microbubbles. However the PRC generated in the current study ‘overshot the mark’ and proved completely resistant to lysis, regardless of treatment.

## Conclusion

Here we sought to create clots more resistant to lysis to increase the rigor of thrombolytic testing. However, this model appears to have gone too far in the other direction and has created clots that are highly resistant to even enhanced lysis with tPA, ultrasound and microbubbles. Sonothrombolysis has previously been shown to be effective in embolic models of stroke with different clot types [51-53] but our study suggests that PRC are particularly resistant to thrombolysis even with the combination of tPA, sonothrombolysis and microbubbles. Our experimental PRC may be representative of clots seen in a subset of stroke patients who are unresponsive to conventional thrombolytic therapies. If so, imaging approaches to identify such clots may allow early pursuit of alternative methods such as mechanical clot retrieval. These clots could also provide a model for development of more rigorous therapies for these patients. Our study highlights the importance of clot structure on the efficacy of thrombolytic therapy and suggests a need for further investigation of *in situ* identification of clot structure to tailor treatment to the patient.

## Additional file

Additional file 1:
**Vascular filling and clot presence (Study 2).** Description of Data: Vessels were perfused post-mortem with Microfil (yellow) to visualise the vasculature and clot presence (black). All animals are presented. (A) shows the vasculature from the view of the circle of willis, (B) shows the corresponding lateral surface of the right hemisphere. Vessels labelled are: middle cerebral artery (MCA), anterior cerebral artery (ACA) and internal carotid artery (ICA). Images of all brains can be viewed in the supplemental data. Treatment groups were saline, tPA or ultrasound insonation with tPA and BR38 microbubbles (U/S + tPA + BR38).

## Abbreviations

JLU: Justus-Liebig University Giessen, Germany
LDF: Laser Doppler Flowmetry
MCA: Middle Cerebral Artery
PRC: Platelet-Rich Clot
rCBF: Regional cerebral blood flow
SAH: Subarachnoid hemorrhage
SD: Standard deviation
SHR: Spontaneously hypertensive rat
tPA: Tissue plasminogen activator
TTC: 2,3,5-triphenyl-tetrazolium chloride
UoN: University of Newcastle, Australia

## References

[1] Hacke W, Kaste M, Bluhmki E, Brozman M, Davalos A, Guidetti D (2008). Thrombolysis with alteplase 3 to 4.5 hours after acute ischemic stroke. N Engl J Med.

[2] National Stroke Foundation (2011). National Stroke Audit - Acute Services Clinical Audit Report 2011.

[3] Rha JH, Saver JL (2007). The impact of recanalization on ischemic stroke outcome: a meta-analysis. Stroke.

[4] Alexandrov AV, Demchuk AM, Felberg RA, Christou I, Barber PA, Burgin WS (2000). High rate of complete recanalization and dramatic clinical recovery during tPA infusion when continuously monitored with 2-MHz transcranial doppler monitoring. Stroke.

[5] Viguier A, Petit R, Rigal M, Cintas P, Larrue V (2005). Continuous monitoring of middle cerebral artery recanalization with transcranial color-coded sonography and Levovist. J Thromb Thrombolysis.

[6] Molina CA, Ribo M, Rubiera M, Montaner J, Santamarina E, Delgado-Mederos R (2006). Microbubble administration accelerates clot lysis during continuous 2-MHz ultrasound monitoring in stroke patients treated with intravenous tissue plasminogen activator. Stroke.

[7] Alexandrov AV, Mikulik R, Ribo M, Sharma VK, Lao AY, Tsivgoulis G (2008). A pilot randomized clinical safety study of sonothrombolysis augmentation with ultrasound-activated perflutren-lipid microspheres for acute ischemic stroke. Stroke.

[8] Molina CA, Barreto AD, Tsivgoulis G, Sierzenski P, Malkoff MD, Rubiera M (2009). Transcranial ultrasound in clinical sonothrombolysis (TUCSON) trial. Ann Neurol.

[9] Alexandrov AV, Molina CA, Grotta JC, Garami Z, Ford SR, Alvarez-Sabin J (2004). Ultrasound-enhanced systemic thrombolysis for acute ischemic stroke. N Engl J Med.

[10] Daffertshofer M, Gass A, Ringleb P, Sitzer M, Sliwka U, Els T (2005). Transcranial low-frequency ultrasound-mediated thrombolysis in brain ischemia: increased risk of hemorrhage with combined ultrasound and tissue plasminogen activator: results of a phase II clinical trial. Stroke.

[11] Roessler FC, Ohlrich M, Marxsen JH, Stellmacher F, Sprenger A, Dempfle CE (2011). The platelet-rich plasma clot: a standardized in-vitro clot formation protocol for investigations of sonothrombolysis under physiological flows. Blood Coagul Fibrinolysis.

[12] Spratt NJ, Fernandez J, Chen M, Rewell S, Cox S, Van Raay L (2006). Modification of the method of thread manufacture improves stroke induction rate and reduces mortality after thread-occlusion of the middle cerebral artery in young or aged rats. J Neurosci Methods.

[13] McLeod DD, Beard DJ, Parsons MW, Levi CR, Calford MB, Spratt NJ (2013). Inadvertent occlusion of the anterior choroidal artery explains infarct variability in the middle cerebral artery thread occlusion stroke model. PLoS ONE.

[14] Murtha LA, McLeod DD, McCann SK, Pepperall D, Chung S, Levi CR (2014). Short-duration hypothermia after ischemic stroke prevents delayed intracranial pressure rise. Int J Stroke.

[15] Dinapoli VA, Rosen CL, Nagamine T, Crocco T (2006). Selective MCA occlusion: a precise embolic stroke model. J Neurosci Methods.

[16] Mishra NK, Albers GW, Davis SM, Donnan GA, Furlan AJ, Hacke W (2010). Mismatch-based delayed thrombolysis: a meta-analysis. Stroke.

[17] Soehle M, Heimann A, Kempski O (2001). On the number of measurement sites required to assess regional cerebral blood flow by laser-Doppler scanning during cerebral ischemia and reperfusion. J Neurosci Methods.

[18] Korninger C, Collen D (1981). Studies on the specific fibrinolytic effect of human extrinsic (tissue-type) plasminogen activator in human blood and in various animal species in vitro. Thromb Haemost.

[19] Nedelmann M, Ritschel N, Doenges S, Langheinrich AC, Acker T, Reuter P (2010). Combined contrast-enhanced ultrasound and rt-PA treatment is safe and improves impaired microcirculation after reperfusion of middle cerebral artery occlusion. J Cereb Blood Flow Metab.

[20] Bederson JB, Pitts LH, Tsuji M, Nishimura MC, Davis RL, Bartkowski H (1986). Rat middle cerebral artery occlusion: evaluation of the model and development of a neurologic examination. Stroke.

[21] Petullo D, Masonic K, Lincoln C, Wibberley L, Teliska M, Yao DL (1999). Model development and behavioral assessment of focal cerebral ischemia in rats. Life Sci.

[22] Almekhlafi MA, Hu WY, Hill MD, Auer RN (2008). Calcification and endothelialization of thrombi in acute stroke. Ann Neurol.

[23] Liebeskind DS, Sanossian N, Yong WH, Starkman S, Tsang MP, Moya AL (2011). CT and MRI early vessel signs reflect clot composition in acute stroke. Stroke.

[24] Marder VJ, Chute DJ, Starkman S, Abolian AM, Kidwell C, Liebeskind D (2006). Analysis of thrombi retrieved from cerebral arteries of patients with acute ischemic stroke. Stroke.

[25] Niesten JM, van der Schaaf IC, Van Dam L, Vink A, Vos JA, Schonewille WJ (2014). Histopathologic composition of cerebral thrombi of acute stroke patients is correlated with stroke subtype and thrombus attenuation. PLoS ONE.

[26] Collet JP, Montalescot G, Lesty C, Soria J, Mishal Z, Thomas D (2001). Disaggregation of in vitro preformed platelet-rich clots by abciximab increases fibrin exposure and promotes fibrinolysis. Arterioscler Thromb Vasc Biol.

[27] Kirchhof K, Welzel T, Zoubaa S, Lichy C, Sikinger M, De Ruiz HL (2002). New method of embolus preparation for standardized embolic stroke in rabbits. Stroke.

[28] Stringer HA, Van Swieten P, Heijnen HF, Sixma JJ, Pannekoek H (1994). Plasminogen activator inhibitor-1 released from activated platelets plays a key role in thrombolysis resistance. Studies with thrombi generated in the Chandler loop. Arterioscler Thromb.

[29] Zhu Y, Carmeliet P, Fay WP (1999). Plasminogen activator inhibitor-1 is a major determinant of arterial thrombolysis resistance. Circulation.

[30] Meairs S, Culp W (2009). Microbubbles for thrombolysis of acute ischemic stroke. Cerebrovasc Dis.

[31] Eggers J, Konig IR, Koch B, Handler G, Seidel G (2008). Sonothrombolysis with transcranial color-coded sonography and recombinant tissue-type plasminogen activator in acute middle cerebral artery main stem occlusion: results from a randomized study. Stroke.

[32] Roessler FC, Oberneyer M, Stellmacher F, Eckey T, Ohlrich M, Royl G (2014). Die THROMBEX–Studie: Korrelation zwischen Thrombushistologie und Outcome thrombektomierter Schlaganfallpatienten [abstract]. E-Book der Neurowoche.

[33] Roessler FC, Teichert A, Ohlrich M, Marxsen JH, Stellmacher F, Tanislav C (2014). Development of a new clot formation protocol for standardized in vitro investigations of sonothrombolysis. J Neurosci Methods.

[34] Busch E, Kruger K, Hossmann KA (1997). Improved model of thromboembolic stroke and rt-PA induced reperfusion in the rat. Brain Res.

[35] Guluma KZ, Lapchak PA (2010). Comparison of the post-embolization effects of tissue-plasminogen activator and simvastatin on neurological outcome in a clinically relevant rat model of acute ischemic stroke. Brain Res.

[36] Okubo S, Igarashi H, Yamaguchi H, Arii K, Sakamaki M, Mizukoshi G (2003). Therapeutic time window of rt-PA on embolic stroke in rats. Int Congress Series.

[37] Tsivgoulis G, Eggers J, Ribo M, Perren F, Saqqur M, Rubiera M (2010). Safety and efficacy of ultrasound-enhanced thrombolysis: a comprehensive review and meta-analysis of randomized and nonrandomized studies. Stroke.

[38] Haelewyn B, Risso JJ, Abraini JH (2010). Human recombinant tissue-plasminogen activator (alteplase): why not use the ‘human’ dose for stroke studies in rats?. J Cereb Blood Flow Metab.

[39] Ciccone A, Motto C, Abraha I, Cozzolino F, Santilli I (2014). Glycoprotein IIb-IIIa inhibitors for acute ischaemic stroke. Cochrane Database Syst Rev.

[40] Uyttenboogaart M, Koch MW, Koopman K, Vroomen PC, De Keyser J, Luijckx GJ (2008). Safety of antiplatelet therapy prior to intravenous thrombolysis in acute ischemic stroke. Arch Neurol.

[41] Fujimoto M, Salamon N, Mayor F, Yuki I, Takemoto K, Vinters HV (2013). Characterization of arterial thrombus composition by magnetic resonance imaging in a swine stroke model. Stroke.

[42] Moftakhar P, English JD, Cooke DL, Kim WT, Stout C, Smith WS (2013). Density of thrombus on admission CT predicts revascularization efficacy in large vessel occlusion acute ischemic stroke. Stroke.

[43] Kirchhof K, Welzel T, Mecke C, Zoubaa S, Sartor K (2003). Differentiation of white, mixed, and red thrombi: value of CT in estimation of the prognosis of thrombolysis phantom study. Radiology.

[44] Eggers J, Koch B, Meyer K, Konig I, Seidel G (2003). Effect of ultrasound on thrombolysis of middle cerebral artery occlusion. Ann Neurol.

[45] Perren F, Loulidi J, Poglia D, Landis T, Sztajzel R (2008). Microbubble potentiated transcranial duplex ultrasound enhances IV thrombolysis in acute stroke. J Thromb Thrombolysis.

[46] Alexandrov AV, Demchuk AM, Burgin WS, Robinson DJ, Grotta JC (2004). Ultrasound-enhanced thrombolysis for acute ischemic stroke: phase I. Findings of the CLOTBUST trial. J Neuroimaging.

[47] Schneider M, Anantharam B, Arditi M, Bokor D, Broillet A, Bussat P (2011). BR38, a new ultrasound blood pool agent. Invest Radiol.

[48] Petit B, Gaud E, Colevret D, Arditi M, Yan F, Tranquart F (2012). In vitro sonothrombolysis of human blood clots with BR38 microbubbles. Ultrasound Med Biol.

[49] Kirchhof K, Sikinger M, Welzel T, Zoubaa S, Sartor K (2004). Does the result of thrombolysis with recombinant tissue-type plasminogen activator (rt-PA) in rabbits depend on the erythrocyte- and fibrin-content of a thrombus?. Röfo.

[50] Overgaard K, Sereghy T, Frellsen M, Pederson H, Hoyer S, Boysen G (1993). Composition of emboli influences the efficacy of thrombolysis with rt-PA in a rat stroke model. Fibrinolysis.

[51] Daffertshofer M, Huang Z, Fatar M, Popolo M, Schroeck H, Kuschinsky W (2004). Efficacy of sonothrombolysis in a rat model of embolic ischemic stroke. Neurosci Lett.

[52] Saguchi T, Onoue H, Urashima M, Ishibashi T, Abe T, Furuhata H (2008). Effective and safe conditions of low-frequency transcranial ultrasonic thrombolysis for acute ischemic stroke: neurologic and histologic evaluation in a rat middle cerebral artery stroke model. Stroke.

[53] Wilhelm-Schwenkmezger T, Pittermann P, Zajonz K, Kempski O, Dieterich M, Nedelmann M (2007). Therapeutic application of 20-kHz transcranial ultrasound in an embolic middle cerebral artery occlusion model in rats: safety concerns. Stroke.

