# Supplementary material for: Platelet rich clots are resistant to lysis by thrombolytic therapy in a rat model of embolic stroke
Source: Exp Transl Stroke Med. 2015 Jan 27;7:2. doi: 10.1186/s13231-014-0014-y (PMC4318170; doi:10.1186/s13231-014-0014-y)
Supplement: Additional file 1: — Vascular filling and clot presence (Study 2). Description of Data: Vessels were perfused post-mortem with Microfil (yellow) to visualise the vasculature and clot presence (black). All animals are presented. (A) shows the vasculature from the view of the circle of willis, (B) shows the corresponding lateral surface of the right hemisphere. Vessels labelled are: middle cerebral artery (MCA), anterior cerebral artery (ACA) and internal carotid artery (ICA). Images of all brains can be viewed in the supplemental data. Treatment groups were saline, tPA or ultrasound insonation with tPA and BR38 microbubbles (U/S + tPA + BR38). [file 13231_2014_14_MOESM1_ESM.pdf]

**Additional File. Vascular filling and clot presence (Study 2).** Vessels were perfused post-mortem with Microfil (yellow) to visualise the vasculature and clot presence (black). All animals are presented. (A) shows the vasculature from the view of the circle of willis, (B) shows the corresponding lateral surface of the right hemisphere. Vessels labelled are: middle cerebral artery (MCA), anterior cerebral artery (ACA) and internal carotid artery (ICA). Images of all brains can be viewed in the supplemental data. Treatment groups were saline, tPA or ultrasound insonation with tPA and BR38 microbubbles (U/S + tPA + BR38).

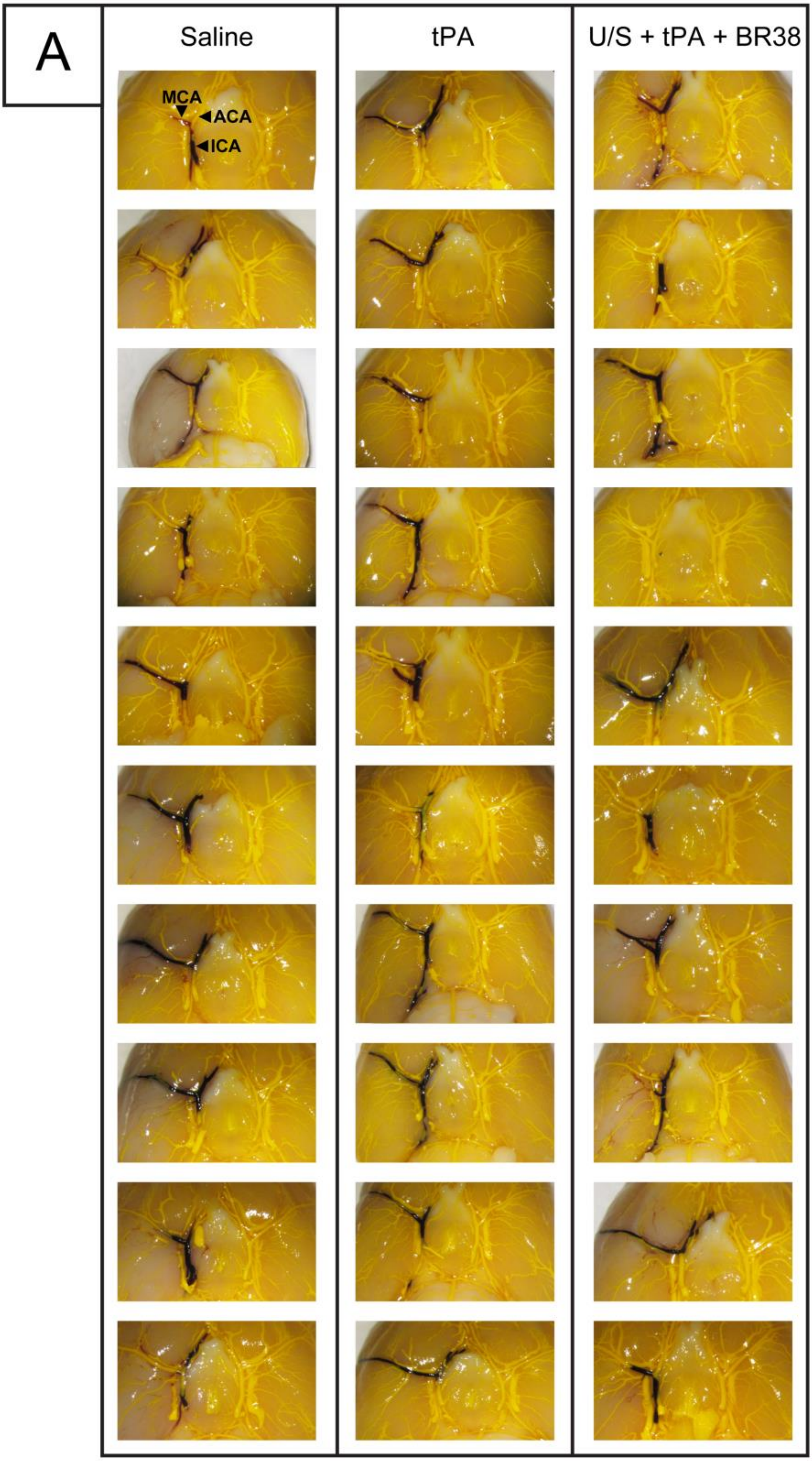
A
Saline
tPA
U/S + tPA + BR38
MCA
ACA
ICA

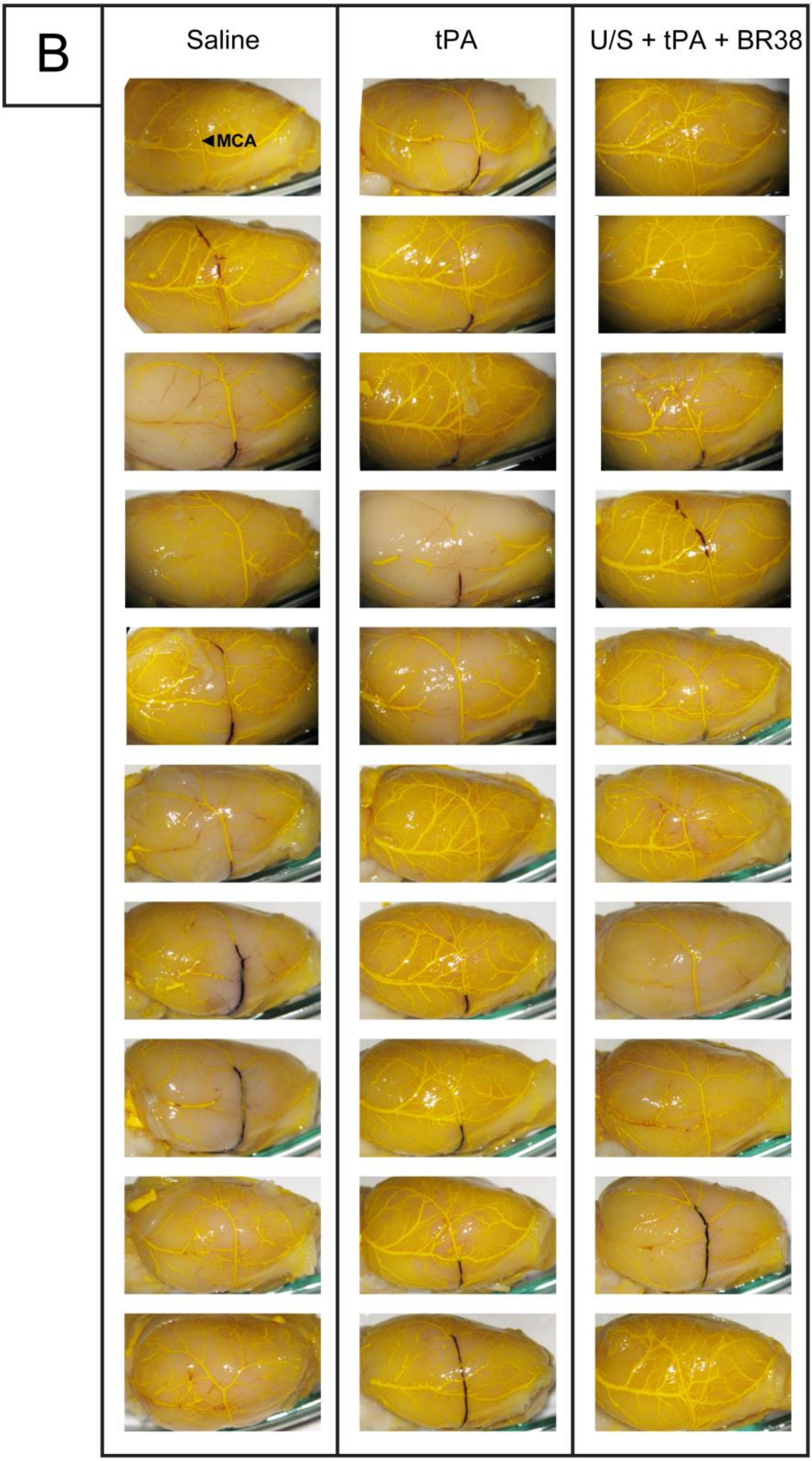
B
Saline
tPA
U/S + tPA + BR38
◄MCA
